# Relation between the Dam’s Weight on Superficial Temperature of Her Puppies at Different Stages of the Post-Partum

**DOI:** 10.3390/vetsci9120673

**Published:** 2022-12-04

**Authors:** Karina Lezama-García, Julio Martínez-Burnes, Juan Carlos Pérez-Jiménez, Adriana Domínguez-Oliva, Patricia Mora-Medina, Adriana Olmos-Hernández, Ismael Hernández-Ávalos, Daniel Mota-Rojas

**Affiliations:** 1 PhD Program in Biological and Health Sciences (Programa de Doctorado en Ciencias Biológicas y de la Salud), Universidad Autónoma Metropolitana, Mexico City 04960, Mexico; 2 Animal Health Group, Facultad de Medicina Veterinaria y Zootecnia, Universidad Autónoma de Tamaulipas, Victoria City 87000, Mexico; 3 El Colegio de la Frontera Sur (ECOSUR), Av. Rancho Polígono 2-A, Ciudad Industrial, Lerma, Campeche 24500, Mexico; 4 Neurophysiology, Behavior and Animal Welfare Assessment, DPAA, Universidad Autónoma Metropolitana (UAM), Unidad Xochimilco, Mexico City 04960, Mexico; 5 Facultad de Estudios Superiores Cuautitlán, Universidad Nacional Autónoma de Mexico (UNAM), Mexico City 54714, Mexico; 6 Division of Biotechnology—Bioterio and Experimental Surgery, Instituto Nacional de Rehabilitación-Luis Guillermo Ibarra Ibarra (INR-LGII), Tlalpan, Mexico City 14389, Mexico

**Keywords:** thermographic image, whelping, dogs, animal perinatology, puppy welfare, newborn puppy

## Abstract

**Simple Summary:**

Newborn puppies have great difficulty achieving a stable temperature, and it has been seen that this is influenced by the weight of the mother. In the present study, the temperature of the puppies was evaluated in eight different areas of the body and at seven different times after birth. It is concluded that the weight of the mothers significantly influences the thermoregulatory capacity of the puppies and that the areas where the lowest puppies’ temperatures were recorded were the thoracic and pelvic limbs, and the highest were the abdominal, thoracic, nasal, and upper left palpebral, especially when newborns dry and at 24 h after birth.

**Abstract:**

The thermal stability of newborns is an essential parameter that can be recorded to evaluate neonatal care. Knowing the thermal windows to evaluate and maintain a constant temperature helps significantly reduce neonatal mortality. This study aimed to assess the superficial temperature alterations in the distinct thermal windows of puppies with mothers of diverse weights and their repercussions. We evaluated the superficial temperature using infrared thermography at eight thermal windows and seven different times: when wet due to the fetal fluid immediately after birth until 24 h of life in newborn puppies from bitches divided into four bodyweight groups. The results revealed a positive correlation between the dam’s weight and the ability to achieve thermostability in the newborn puppies in all the evaluated thermal windows. The time effect showed the lowest temperatures when the puppies were still wet, a gradual increase, and the highest temperature at 24 h after birth. The thermal windows with the highest temperatures were abdominal, thoracic, nasal, and upper left palpebral, and those with the lowest were the thoracic limb brachial biceps, thoracic limb elbow, metacarpal, and femoral pelvic limb. A significant increase in the temperatures in the thermal windows of the abdominal, thoracic, and upper left palpebral immediately after ingesting colostrum was observed. The weight of the dams is an important factor that positively intervenes in the thermoregulatory capacity of the puppies, especially when newborns are dry and have been passed 24 h after birth.

## 1. Introduction

Birth is a great challenge for the newborn since it must adapt to extrauterine life and survive the neonatal period. Low vitality is a recurrent issue in veterinary perinatology, and several factors can culminate in newborn death [1]. Various studies on neonate mammals demonstrate that maternal behavior directly impacts newborn survival [2,3,4]. Moreover, mortality rates between birth and weaning can be linked to maternal and newborn behavior [1,5,6,7,8]. Other than this, neonatal viability is related to fetal maturity, the environmental circumstances, and maternal care [9]. In dogs (*Canis familiaris*), mortality in newborn puppies varies from 5 to 35% [10]. Among several maternal, pregnancy, and newborn-related factors, hypothermia is considered to be a condition that could adversely affect newborn survival [11]. One of the main parameters to be controlled during birth is thermal stability that is a fundamental aspect of neonatal care, and maintaining a constant temperature helps significantly reduce neonatal mortality [12]. In other words, thermoregulation plays an essential role in the survival of all altricial species and poikilothermic organisms, especially in neonates [13,14,15,16,17]. Individuals that make the transition from the warm environment of the uterus to an extrauterine environment cause a significant decrease in their corporal temperature at birth [18,19], considering that 35–37 °C is the normal rectal temperature in newborn puppies of less than 1 week [20,21]. This thermal change impacts the newborn pup’s ability to thermoregulate since mechanisms such as the shivering reflex and vasoconstriction are still underdeveloped [22,23].

Various methods measure temperature in animals, but most can be invasive, altering the final value [24,25]. This can be due to the stress of being manipulated [26,27]. For this reason, in recent years, it has been seen that a non-invasive technique to evaluate the temperature changes, both on farms and in companion animals, is infrared thermography (IRT) [28]. IRT can also record the exact measurements of the body surface temperature of any organism from a distance of 30 cm or more [29]. This technique detects changes in the blood flow of the microvasculature in response to pathophysiological or environmental events such as heat or cold stress [30].

Studies have been carried out on sheep [5,31,32] and in pigs [33,34] where it has been seen that the survival of the newborn can be influenced by elements such as the dam weight and body condition, parity, breed, age, newborn’s weight at birth, and the litter size, as well as by the maternal and offspring behavior [2,35]. In dogs, studies have evaluated the effect of the dam’s weight on the puppy’s weight at birth, the litter size, vitality, and the puppy’s survival [10,36,37,38]. Additionally, the effect of the dam’s weight on the presentation of asphyxia and newborn hematological values were evaluated [39]. In some species, newborns from larger females have reported less thermoregulatory problems, and a higher temperature in central zones of the body [12,13,14,15,16,17,25,26], so similar results can be expected in dogs. However, there is still no information on thermal windows in puppies (e.g., ocular or auricular regions), known as a body region of the neonate where superficial temperatures can be assessed [40]. The use of these regions could help understand the process of thermoregulation in newborn dogs, and thus help reduce the high mortality rates in this species. In addition, the effect of the dam weight and the pups on their thermoregulatory capacity during the first 24 h after birth is also unknown.

For this reason, this study aims to evaluate the alterations in the different thermal windows of puppies with mothers of different body weights and their repercussions on the presentation of hypothermia in newborns. The present authors have the following hypothesis: (1) newborns from bitches with a high body weight will have fewer thermoregulation problems, and those from low body weight bitches will have more thermoregulation complications; in the same way, the body weight of puppies can affect thermoregulation due to the large body surface that they present; (2) thermal windows with more elevated temperatures will be the ones closest to the brain and vital organs (palpebral, thoracic, abdominal, and nasal); and (3) the thermal windows with the lowest temperatures will be the ones furthest from the vital structures (thoracic limbs and pelvic limbs).

## 2. Materials and Methods

### 2.1. Facilities

A network of 10 participating veterinary clinics was formed to monitor canine and puppy births. The study region is located in southeastern Mexico, specifically in the Yucatan peninsula, bordering to the north and northeast with Yucatan, to the east with Quintana Roo, to the south with Guatemala and Belize, to the west with the Gulf of Mexico, and to the southwest with Tabasco. The owners of pregnant bitches were offered medical attention for prenatal control from day 25 of pregnancy to the first 48 h after parturition.

### 2.2. Study Population

Seventy-two pregnant young multiparous bitches (2–4 births) were recruited. However, 12 bitches required an emergency C-section during parturition and were excluded. In total, 290 puppies from 60 parturient bitches were included in the present study. Within the breeds included in this study we found Chihuahua, German Shepherd, Labrador, Golden Retriever, Great Dane, Standard Schnauzer, Cocker Spaniel, Poodle, Scottish Terrier, and Belgian Shepherd. A total of 60 bitches were divided into 4 groups of 15 bitches each, according to their body weight as follows: G_1_ (4–8 kg) *n* = 47 puppies, G_2_ (8.1–16 kg) *n* = 68 puppies, G_3_ (16.1 to 32 kg) *n* = 79 puppies, and G_4_ (32.1 to 39.6 kg) *n* = 96 puppies. The obstetric condition of the bitches, as well as the gestation from the beginning to the term (from day 28 to day 30 after mating), was monitored as reported later in the prenatal procedure section.

The body weight of the bitches was obtained using a digital scale from Salter Weight Tronix Ltd., West Bromwich, UK, immediately at the first stage of whelping, when the contractions started. The inclusion criteria were: (a) clinically healthy dogs with valid vaccination/deworming records; (b) no clinical records of reproductive problems; and (c) bitches with ultrasonographic and radiographic studies to sustain a natural birth. The exclusion criteria were: (a) bitches with previous cases of dystocia or pyometra; (b) primiparous dams; (c) malformed fetuses; (d) the administration of birth inducers or accelerators; (e) behavioral problems (aggressive females); (f) bitches with a body condition over 8 (obese) as per the WSAVA scale [41]; (g) any large breeds and brachycephalic bitches due to their reported high incidence of dystocia; and (h) those that required an emergency C-section [42]. The bodyweight ranges were based on the Federation Cynologique Internationale (FCI) [43].

### 2.3. Clinical History

The clinical history of the sampled animals was carried out by collecting data such as their age, type of diet, parity number, bodyweight, breed, history of preventive medicine, and description of where they lived. These data were collected with diagnostic and monitoring methodologies controlled by the veterinary software SmartZooft^®^ LAN, 14 K version, developed by SQUENDA^®^, Mexico City, Mexico.

### 2.4. Prenatal Procedures

The pregnancy diagnosis was carried out from days 28 to 30 post-mating in the bitches, using a Mindray^®^ model DP-30VetPower ultrasonography equipment (Shenzhen, China) with Doppler and Pulsed Doppler (PW) advanced technologies, equipped with a 3.5 MHz convex transducer. The gestation was corroborated by visualizing the gestational sacs and the presence of a heartbeat in the embryos. The following ultrasonographic assessment was carried out between days 40 and 43 of gestation to determine the viability of the fetuses and their growth and health status. Subsequently, X-ray studies were carried out between days 48 and 50 to determine the number of fetuses and their head sizes and to make measurements to predict if there could be a dystocic whelping, which could even end in cesarean section due to some cephalopelvic disproportion [44]. From day 60 post-mating, the bitches and fetuses were monitored using a Sonolife^®^ (Chihuahua, Mexico) brand antepartum monitor, model Smart Monitor Color, with a multi-crystal pulsed Doppler transducer to evaluate the health status of both the mother and the fetuses, uterine activity, number, duration, interval and frequency of the contractions, and the fetal heart rate, following a methodology previously reported in piglets by other authors [45]. In cases where fetal heart rate decelerations type 2 (DIP 2) arose (a drop in the fetal heart rate that begins after the onset of a uterine contraction and returns to the baseline only after the uterine contraction has ended, caused by uteroplacental insufficiency), an emergency cesarean section was performed, and these bitches were excluded from the group, that is why none of the puppies used for this study presented meconium-stained amniotic fluid. In the same way, the monitoring of the vital signs of the dams was carried out using a veterinary monitor DESEGO^®^ (Mexico City, Mexico) model M8i SVGA to evaluate the electrocardiographic tracings, respiratory rate, oxygen saturation, temperature, and blood pressure from the probable date of whelping. However, the bitches were only hospitalized in cases where delivery care took place in hospital/clinic facilities and not when delivery took place at the bitches’ homes.

### 2.5. Puppies

Once parturition started, 290 puppies were evaluated when expelled and the bitch began to lick them to separate them from the membranes. The temperature of the room where the bitches gave birth was not controlled because the study was carried out in different clinics, hospitals, or even in the homes of the animals. However, in the city where the study was carried out, the climate is tropical, and the temperatures oscillate between 36 and 40 °C. In all cases, when parturition started, the air conditioners and fans were turned off. The temperature measurements in the puppies were recorded in 7 different stages: (1) wet puppy (wet) with amniotic fluid, once the dam released the puppy of the membranes and temporarily stopped licking it; (2) dry puppy (dry), which was dried by rubbing for 1 min with rag towels and immediately afterwards, the puppy was returned to the mammary gland area of the bitch, and until the puppy made contact with the teat on its own; (3) colostrum pup (colostrum), immediately after it ingested colostrum and separated from its mother’s teat; (4) at 30 min of birth (30 min AB); (5) at the first hour of birth (1 h AB); (6) at 4 h after birth (4 h AB); and (7) at 24 h after birth (24 h AB). It is worth mentioning that all the temperatures were obtained without manipulating the puppies except to dry and weight them and to evaluate their vitality.

Finally, the weight of the puppies at birth was obtained using a digital scale from Salter Weight Tronix Ltd., West Bromwich, UK, after drying them. The puppies’ weight was obtained only once when the bitch stopped licking the placental membranes. All the puppies of each litter were evaluated. For their identification we used a quick-drying indelible ink marker. It is worth mentioning that the puppies were only fed milk from the dam and were not supplemented with additional milk formulas, and the puppy was returned to the area of the mammary gland so that it could start suckling itself.

### 2.6. Infrared Thermography

**Figure 1:**
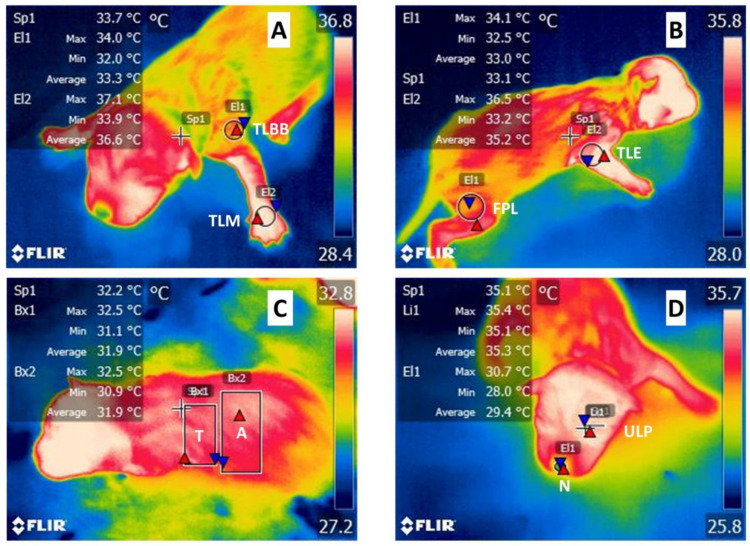
Thermal windows in newborn puppies. (**A**) Thoracic limb brachial biceps (TLBB) and thoracic limb metacarpals (TLM) windows were obtained by placing circular figures from the area where the armpit begins to half the width of the thoracic limb and in the joint formed by the metacarpals covering the area from medial to lateral end, respectively. (**B**) Thoracic limb elbow (TLE) and femoral pelvic limb (FPL) windows were obtained by placing circular figures in the elbow area covering the vertex formed by the humerus-radio-ulnar joint and in the space bounded by the edge of the pelvic limb in the biceps femoris region, respectively. (**C**) Thoracic (T) window was made with rectangular figures delimited by the axillary area, the area of the last rib, and from the region of the spinal vertebrae and to the ventral part of the abdominal region (A) window was delimited by two millimeters after the last rib, to the inguinal area, and from the region of the spinal vertebrae and to the ventral part of the abdomen. (**D**) Nasal (N) and upper left palpebral (ULP) windows were made with circular figures delimited by the edges of the nasal mucosa and at the area of the edge of the left upper eyelid, respectively. Sp1: Spot 1; El1: Ellipse 1; El2: Ellipse 2; Bx1: Box 1; Bx2: Box 2; Li1: Line 1; red triangles maximum temperature of that zone; blue triangles minimum temperature of that zone.

A total of 16,240 thermographic data were evaluated with their minimum, average, and maximum values. These data resulted from 290 puppies, from which 3 thermograms were taken: one from the facial area, another from the left lateral, and another from the right lateral regions. The facial thermogram included the thoracic limbs to not only record the nasal (N) and upper left palpebral (UPL) temperature but also to have a frontal image of the thoracic limb metacarpals (TLM) region. Likewise, the thermogram taken from the right lateral region recorded the thoracic limb elbow (TLE) and femoral pelvic limb (FPL) The temperature records of the thorax and abdomen were obtained from the left lateral image. In each puppy, 8 thermal windows were identified at 7 different times, which are explained in detail in Figure 1; the thoracic limb brachial biceps (TLBB) and thoracic limb metacarpals (TLM) windows were obtained from the area where the armpit begins to half the width of the thoracic limb to the joint formed by the metacarpals, covering the area from the medial to lateral end, respectively. The thoracic limb elbow (TLE) and femoral pelvic limb (FPL) windows were delimited from the elbow area covering the vertex formed by the humerus-radio-ulnar joint to the space bounded by the edge of the pelvic limb in the biceps femoris region, respectively. The thoracic (T) window was delimited by the axillary area, the anatomical position of the last rib, and from the region of the spinal vertebrae to the ventral part of the abdominal region (A). This window was delimited by two millimeters after the last rib to the inguinal area, and from the region of the spinal vertebrae to the ventral part of the abdomen. The nasal (N) and upper left palpebral (ULP) windows were delimited by the edges of the nasal mucosa and at the edge of the left upper eyelid, respectively.

Thermographic images were obtained with an infrared camera FLIR^®^ model Thermal TM E80, FLIR Systems, Wilsonville, OR, USA, with the following specifications: IR resolution 320 × 240 pixels, thermal sensitivity < 0.045 °C, accuracy ± 2 °C or 2% of reading in the ambient temperature of 10 °C to 35 °C and image frequency of 60 Hz. All the images were collected with an emissivity of 0.95 at a uniform distance of 30 cm. Thermographic images were taken to evaluate 8 different zones: (1) thoracic limb brachial biceps (TLBB); (2) thoracic limb elbow (TLE); (3) thoracic limb metacarpals (TLM); (4) femoral pelvic limb (FPL); (5) thoracic (T); (6) abdominal (A); (7) nasal (N); and (8) upper left palpebral (ULP). The thermographic images were saved in JPEG format to analyze later using specialized s FLIR Tools software^®^ 6.x (FLIR Systems, Wilsonville, OR, USA). The maximum, minimum, and mean temperature of each thermal window was obtained in each of the 7 different stages.

It is important to mention that to avoid the type of floor where the puppy is expelled, which could influence the gain or loss of heat, a thermosetting mat based on foam rubber (ethyl vinyl acetate) with a surface area of 1 m^2^, 1 cm deep, with a weight of 0.032 kg, and a matte finish was used in all cases.

### 2.7. Statistical Analysis

Descriptive statistics were obtained for all the variables examined following the procedure outlined in the two-factor Analysis of Variance (ANOVA StatSoft Inc. 0.8, Tulsa, Oakland, CA, USA) to compare the effects of the dam’s bodyweight groups (4 categories: G_1_, G_2_, G_3_, and G_4_) and time (7 categories: wet, dry, colostrum, 30 min, 1 h, 4 h, and 24 h) by eight different zones (TLBB, TLM, TLE, FPL, T, A, N, and UPL). Additionally, a three-factor ANOVA was used to compare the effects of the dam’s body weight (4 categories: G_1_, G_2_, G_3_, and G_4_), time (7 categories: wet, dry, colostrum, 30 min, 1 h, 4 h, and 24 h) and zones (8 categories: TLBB, TLM, TLE, FPL, T, A, N, and ULP). The Tukey test (*p* < 0.05) showed a contrast of means. Spearman’s rank test was used to establish the correlation between the variables and the relationship between the temperatures and the dam’s weights.

### 2.8. Ethical Statement

All the owners of the study animals were asked to sign their informed consent to carry out the procedures. All work was performed under the guidelines and lineaments of Mexico’s Official Norm NOM-062-ZOO-1999 on the technical specifications for animal production, care, and ethical use in applied ethological studies [46]. The Ph.D. Program in the Biological and Health Science Academic Committee approved this project with approval number CBS.114.19. The animals included in the present study were treated gently and were not touched or stressed, since infrared thermography is a non-invasive technique. The only time the puppies were taken was when they were finished drying; they were weighed and their vitality was assessed, and this procedure did not take more than 2 min.

## 3. Results

### 3.1. Infrared Thermography

**Figure 2:**
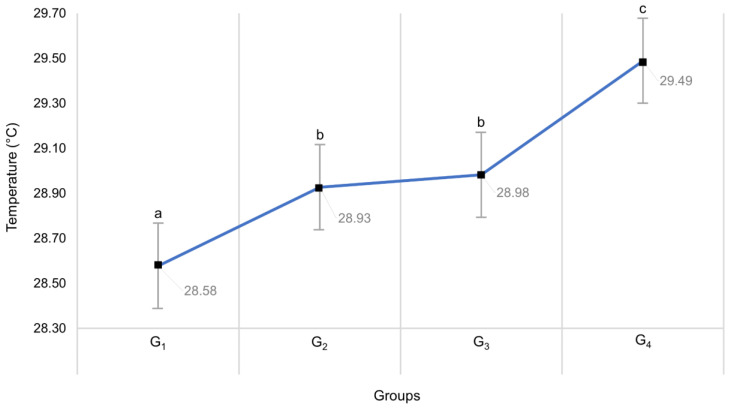
Mean ± standard error of the temperatures in the 4 groups of bitches. Different letters ^(a,b,c)^ indicate significant differences in temperatures between the 4 groups of bitches (G_1_, 4–8 Kg; G_2_, 8.1–16 Kg; G_3_, 16.1–32 Kg; G_4_, 32.1–39.6 Kg) and in the 7 measurement times.

The standard error (SE) of the mean of the eight thermal windows, including the different puppies’ time-point, was analyzed between the four groups of bitches. As seen in Figure 2, there are statistically significant differences (*p* < 0.0001) between the four groups (G_1_, G_2_, G_3_, and G_4_), but in general and in all thermal windows, groups G_2_ and G_3_ were similar.

**Figure 3:**
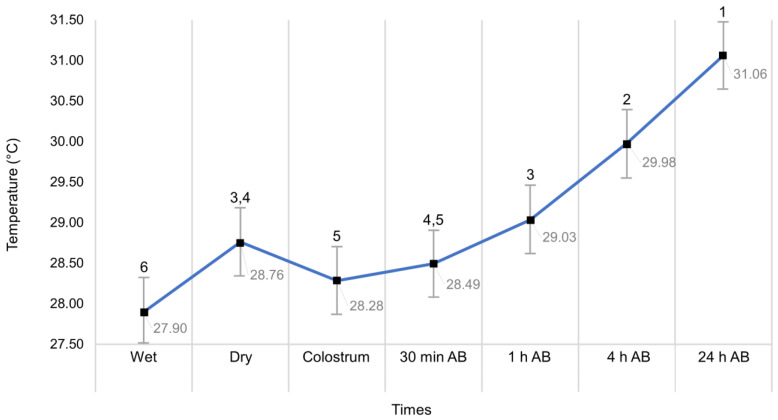
Neonatal average temperature values throughout the experimental period regardless of the surface region or dam’s weight. Means ± standard errors. Different numbers ^(1,2,3,4,5,6)^ indicate significant differences between times in the same dam’s weight group. AB: after birth.

In the same way, the evidence indicates that there were significant differences between the temperature means of the thermal windows and the different puppies’ evaluation times (wet, dry, colostrum, 30 min AB, 1 h AB, 4 h AB, and 24 h AB), as can be seen in Figure 3.

**Figure 4:**
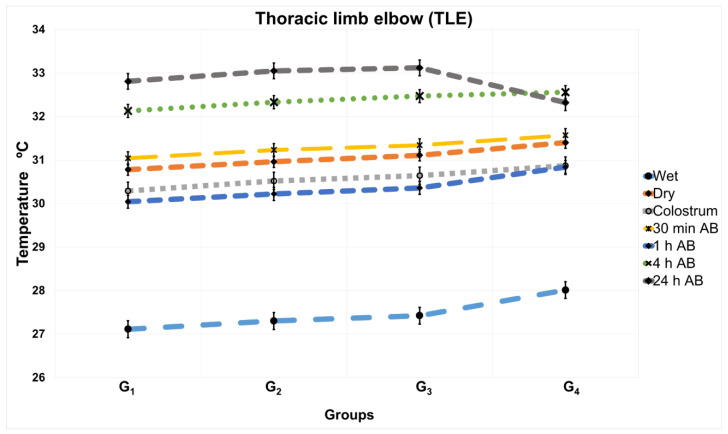
Interaction between group and time in thoracic limb elbow (TLE) thermal window at different times in newborn puppies from bitches classified into 4 groups according to their weight. G_1_, dams between 4 and 8 Kg; G_2_, dams between 8.1 and 16 Kg; G_3_, dams between 16.1 and 32 Kg; G_4_, dams between 32.1 and 39.6 Kg, AB: after birth.

There was no interaction between the groups and times (*p* > 0.05), except on the thoracic limb elbow (TLE) thermal window, in which there was an interaction between the group and time (*p* < 0.0001), as it can be observed in Figure 4.

On the three-way ANOVA results (the group, thermal window, and time), there were differences between the groups (*p* < 0.0001), between the thermal windows (*p* < 0.0001), and differences between the times (*p* < 0.0001). There was an interaction between the group and thermal window (*p* < 0.0001), there was no interaction between the group and time (*p* > 0.05), there was an interaction between the thermal window and time (*p* < 0.0001), and there was no interaction between the three factors (the group, thermal window, and time) (*p* > 0.005).

### 3.2. Effect of Maternal Weight and Litter Size on Neonatal Superficial Temperature Changes

The highest temperatures were recorded in puppies born to bitches from the G_4_ group, and in all the thermal windows evaluated, the lowest temperatures were in G1 bitches. Regarding G_2_ and G_3_, similarities were observed between them (Table 1, Table 2, Table 3, Table 4, Table 5, Table 6, Table 7 and Table 8). The average litter size for G_1_ was 3.13 puppies, for G_2_ was 4.4 puppies, for G_3_ was 5.2 puppies, and finally for G_4_ was 6.4 puppies. The average of the puppies’ weight for each group was G_1_ 195.12 g, G_2_ 233.31 g, G_3_ 332 g, and G_4_ 396.65 g. It is also important to mention that the mean gestational age by groups was G_1_ 58 days, G_2_ 61 days, G_3_ 60 days, and G_4_ 67 days.

### 3.3. Effect of Colostrum Consumption on Thermoregulation

Once the pups ingest colostrum, the temperature decreased significantly *(p <* 0.0001) at windows TLBB, TLE, TLM, FPL, and N. For example, in the TLBB window (Table 1) and TLE (Table 2), the range of this decrease was 0.48 °C in all the groups (G_1_, G_2_, G_3_, and G_4_). For the TLM window (Table 3), the average of this decrease was 0.85 °C. However, in thermal window T, the temperature increased immediately after the ingestion of colostrum by an average of 0.21 °C in the four groups (Table 4). The same occurred in thermal window A, with an average of 0.14 °C above the temperature of dry puppies (Table 5). On the FPL window (Table 6), the decrease was 0.55 °C; for the N window (Table 7), the decrease range was 0.29 °C. Similarly, the temperature in the ULP thermal window increased immediately after the pups ingested colostrum with an average of 1.61 °C above the dry stage (Table 8).

### 3.4. Changes in Temperature according to the Regions Evaluated

The thermal windows with the lowest temperatures recorded were the thoracic limb metacarpals (TLM) in wet puppies (26.98 ± 0.073) (Table 3) and the femoral pelvic limb (FPL) thermal window in wet puppies (27.54 ± 0.071) (Table 6). The thermal windows with the highest temperatures recorded were the abdominal (34.23 ± 0.047) at 24 h after birth (Table 5) and the upper left palpebral (ULP) (34.07 ± 0.044) at 24 h after birth (Table 8).

### 3.5. Thermal Response of the Newborn Due to the Effect of Time

The data suggest that it is more difficult for wet puppies to reach thermostability. The lowest temperatures were observed when the puppies were humid (26.98 ± 0.073) in the thermal window TLM (Table 3), and the highest were observed 24 h after birth (34.23 ± 0.047) in the thermal window A (Table 5).

The findings reflected in Table 1 indicate that the temperatures of the puppies in the TLBB thermal window at 7 different times (wet, dry, colostrum, 30 min AB, 1 h AB, 4 h AB, and 24 h AB) show statistically significant differences (*p <* 0.0001) between the groups of the neonates of the bitches from the G_1_ and G_4_ groups. Observing a 0.86 °C difference between the temperatures recorded in the puppies born to small-sized bitches (G_1_) when they were still wet, with the puppies born from large-sized bitches (G_4_) when they were also still wet, showing this same difference of 0.86 °C when compared to the G_1_ group with G_4_ at 24 h AB according to descriptive statistics. When comparing the temperatures of the wet puppies of the G_1_ group with those recorded in the G_4_ group at 24 h AB, a difference of 4.02 °C was recorded, the broadest f differences between the temperatures evaluated in this thermal window. There was no interaction between the group and time (*p* > 0.05).

Regarding the temperature of the puppies at the different times, the evidence indicates that there is a statistically significant difference (*p* < 0.0001) in the temperature of the TLE region between the puppies born to bitches that are included in group G_1_ and the puppies born to bitches in G_4_. Statistical differences among the timepoints were observed in all four experimental groups. For puppies evaluated at 24 h AB, no significant differences were registered between G_1_, G_2_, and G_3_, but with differences when compared to G_4_, which indicates that the surface temperature of the puppies in the TLE region tends to stabilize and reaches the higher temperature values in puppies from heavy weight bitches.

Regarding the puppies born to bitches of a lower weight (G_1_), there were statistically significant differences (*p* < 0.0001) in the TLE temperatures between the wet times and 24 h AB in a range of 4.6 to 5.7 °C. It is also essential to observe that at the TLBB window (Table 1) and this thermal window (Table 2), the range of the decrease in the temperature immediately after consuming colostrum (between 31.04 °C and 31.57 °C) was roughly 0.5 °C in all groups (G_1_, G_2_, G_3_, and G_4_). There is an interaction between the group and time (*p* < 0.0001), as it can be observed in Figure 4. That is to say that there is an interaction between the groups and times, specifically in G_4_ where there is an overlap of the temperature values for time 4 h AB and 24 h AB and also for time 1 h AB and colostrum time.

Like the previous tables, this thermal window (TLM) (Table 3) repeats that the lowest temperatures were recorded in the puppies born to the G_1_ bitches, and the highest temperatures in the puppies born to the G_4_ bitches. It is worth mentioning that the lowest temperature of this study (26.98 ± 0.073) was recorded in this thermal window in the wet puppies of the G_1_ group. Another important piece of data to point out in this table is that the decrease in the temperature once the pups ingested colostrum was statistically significantly different *(p* < 0.0001) between the dry and colostrum time, showing an average of 0.85 °C less than in the dry time in all groups. There was no interaction between the group and time (*p* > 0.05).

Table 4 shows, unlike the previous tables, the most crucial point to remark, that the T window temperature of the puppies increased immediately after the ingestion of colostrum by an average of 0.21 °C in the four groups. Furthermore, it is observed that at all times, the temperature difference between group G_1_ and G_4_ was 0.5 °C, except in wet and dry times, in which the difference between these groups was 0.41 °C and 0.49 °C, respectively, showing statistically significant differences (*p* < 0.0001). There was no interaction between the group and time (*p* > 0.05).

Notably, the A temperature of newborn puppies from the G_1_ at dry was 0.52 °C lower than in G_4_. At the same time, the average temperature of the puppies in G_1_ at 24 h AB was 0.49 °C lower than in G_4_. This effect shows that the dam’s weight influenced the thermal response at the abdominal level of newborn puppies at all the time-points.

When comparing the different times per group, the puppies from G_1_ had a significant statistical difference (*p* < 0.0001) between the A temperature registered at wet and dry (an average difference of 2.76 °C), at 4 h (+3.73 °C when compared to wet values), and at 24 h AB, with an average increase in the A temperature of 4.27 °C for the G_1_. Similarly, in G_2_, G_3_, and G_4_, the A temperature at 24 h AB increased by approximately 4.24 °C and was statistically significant when comparing the wet time in all the groups. An increase in the A temperature can also be observed between dry and colostrum times, with an average of +0.14 °C. It is also worth mentioning that the highest temperatures in all the studies were recorded on this thermal window at 24 h AB in the puppies born to bitches of group G_4_. There was no interaction between the group and time (*p* > 0.05).

When comparing the average temperature of FPL in the four groups, significant statistical differences (*p* < 0.0001) can be observed between G_4_ and the other three groups at all the measured times; the newborn puppies from G_4_ had higher temperatures and a mean difference of 0.78 °C when compared to G_1_, particularly at 30 min AB (mean difference of 0.86 °C) and at 4 h AB (mean difference of 0.81 °C).

Within the groups, a statistically significant difference was found in all the groups at 4 h AB and 24 h AB (*p* < 0.0001). In G_1_, from the wet time to 4 h AB, the puppies from low-weight dams had an increase in the FPL temperature average of 2.08 °C, while the temperature from wet to 24 h AB increased by an average of 3.16 °C. A similar case was observed in G_2_, G_3_, and G_4_ at wet and 24 h AB (3.16, 3.18, and 3.17 °C, respectively). Additionally, wet to 4 h showed similar increasing temperatures as those registered in G_1_, 2.22 °C for G_2_, 2.11 °C for G_3_, and 2.14 for G_4_. There was no interaction between the group and time (*p* > 0.05).

The average temperatures of the N window show a significant statistical difference (*p* < 0.0001) between G_1_ and G_4_, differing from the first time of evaluation (wet) by 0.96 °C and at 24 h AB by 0.93 °C.

The differences between the temperatures of N taken at the seven times per group show that in all the groups, the temperatures at wet were significantly different (*p* < 0.0001) from the rest of the evaluated times, with a mean increase in the temperature from wet to dry in the four groups of 5.86 °C. There was no interaction between the group and time (*p* > 0.05).

The mean ULP temperatures of G_4_ have statistically significant differences (*p* < 0.0001) from the other three groups, except for G_2_ during the dry period. When comparing the average temperature of G_4_ in all the measured times (32.79 °C) to the same average values in G_1_, G_2_, and G_3_, in all the times, the IRT temperatures were higher in G_4_, with a mean temperature difference of +0.42 °C, +0.37 °C, and +0.29 °C, respectively.

A statistically significant difference (*p* < 0.0001) was recorded within all the groups at wet and 24 h AB.

For G_1_, the temperature increased from the first evaluation to 24 h AB at an average of 2.93 °C; for G_2_, it increased by 2.87 °C, G_3_ by 2.96 °C, and G_4_ 2.98 °C. Interestingly, when considering the difference between the times dry and colostrum, the newborn puppies ULP average temperatures in all the groups increased by 1.61 °C as an effect of the colostrum intake.

Subsequently, the correlations between the mother’s weight and the temperatures of the pups at different times and in the different thermal windows were evaluated using Spearman’s rank correlations. Table 9, Table 10, Table 11 and Table 12 report the existing correlations between the weight of the dam and the temperature of the puppies, where the weights of the four groups (G_1_, G_2_, G_3_, and G_4_) and their respective temperatures by the time and thermal window indicate how the temperature tends to vary at different times and in different thermal windows. In almost all cases, there was a positive correlation with statistically significant differences (*p* < 0.0001) (Table 9, Table 11 and Table 12). These results mean that the higher the weight of the bitches, the higher the temperatures recorded in the pups were. Only one case was observed where the correlation was negative (Table 10), in the thermal window TLE at 24 h AB. In this case, as the weight of the dams increased, the temperature of the puppies decreased in the TLE window. The r values in all tables were between 0.127 and 0.634, except in Table 10 of the TLE thermal window, where *r*= −0.177 (*p* = 0.0026) at 24 h AB. The highest correlations were observed in the Table 9 TLBB window (colostrum *r =* 0.601) and Table 11 TLM window (colostrum *r* = 0.634). There was no interaction between the group and time (*p* > 0.05).

## 4. Discussion

The present study assessed the superficial temperature at eight anatomical regions or thermal windows and at different times in newborn puppies born from bitches with distinct weights. According to the results, there is an association between the surface temperature of the pups and the weight of the dams, having the highest temperature in all the thermal windows at all the evaluation times in G_4_ (bitches with the highest weight range of 32.1–39.6 kg). Additionally, although the temperatures at the wet and dry times differed between the four groups, both had the lowest values in all groups. At 24 h AB, the temperature in all the thermal windows showed a correlation with, regardless of the weight of the dam, similar values, meaning that the thermoregulatory capacity of the puppies after 24 h may depend on the dam’s weight. It is also important to mention that the rectal temperature of the puppies or the bitches was not recorded, to avoid an invasive approach to temperature evaluation.

This study’s results show the effect of the dam’s weight on the superficial temperature of canine neonates. The statistical analyzes carried out in this study revealed that the thermoregulation capacity of the puppies is closely linked to the weight of the bitches, since significant differences (*p* < 0.0001) between the temperatures recorded in all the groups of bitches were observed. Newborns from low-weight bitches had lower temperature values for all the thermal windows at every measured time than puppies from the heaviest bitches. This thermoregulatory dependence on the weight and time has also been reported in dog puppies that cannot stabilize their temperature rhythm for several days after birth, but their rectal temperatures become stable six weeks after birth [47]. It also has been reported that dogs of a small size tend to lose heat quickly because they have a bigger surface area to volume ratio [48]; therefore, they require a greater heat production to maintain their thermostability [49]. Additionally, it can be attributed to the wide variability of canine breeds [49]. For example, short-haired dogs from breeds like Miniature Pinscher, pit bull mix, or pointer hound mix presented a higher surface temperature of approximately 2 °C more than long-haired breeds such as Shih-tzu, miniature schnauzer, Labrador mix, or Pomeranian dogs [49]. Due to this, the environment’s temperature where the puppies live could have a favorable or unfavorable effect on their welfare [50]. In addition, their weight impacted their health and vitality [51]. Veronesi [52] mention that the body size of a breed also influences the viability of newborn puppies, assessed with Apgar score systems, where small-sized puppies may have the highest levels of distress but higher chances of survival when compared to large-sized animals.

Birth weight is essential for the survival of neonates, and it is one of the most important factors that influences temperature loss since the body mass index (BMI) determines the capacity of thermoregulation in newborns like foxes [53]. Regarding the dam’s body weight, this depends on the fetus’s presence (representing 1 to 3% of the total weight of the bitch), and this bodyweight also influences the birth weight of newborns [54]. In addition, according to Mila et al. [37], the birth weight is influenced statistically significant by litter size, presenting a higher number of low-birth-weight puppies in large litters compared to small litters. According to the data obtained in this study, it was observed that the larger the litter size and the greater the weight of the puppies at birth, the more easily stabilized thermoregulation was in the puppies that came from larger litters and had heavier weights raised at birth.

Attempts have been made to establish the most appropriate thermal regions or windows that provide better information on the thermal changes in veterinary medicine [55]. Thus, thermal utility windows are lacrimal caruncle, eye, ear, thorax flanks, appendicular area, and face [13,40,56]. However, other thermal windows have also been suggested in pathological cases, such as the mammary gland and the ventral window for cases of mammary gland cancer and the neck for cases of hypothyroidism in cats [57,58].

The most notable thermoregulatory changes in newborn puppies were low temperatures in the distal regions, that is, in the thermal windows of the pelvic and thoracic limbs (TLM and FPL), and the highest temperatures in the thermal windows A, T, and ULP. The peripheral and central circulation could explain these findings. The most important structures related to the metabolism and vital functions are in the thoracic, abdominal, and cranial regions [40]. The appendicular windows presented the lowest temperatures. This can be explained by the limited locomotion of altricial species and their inability to walk or stand up immediately after birth. However, newborn puppies can crawl and have active movement of the limbs, although these movements are minimal and may not promote significant thermal changes detected with IRT because neonates have a low blood pressure and, thus, the blood flow in the periphery is lower than the central circulatory system [16]. When considering the thermographic changes in the skin of newborn dogs, it is important to highlight the degree of neurological development responsible for the mechanism of thermal modulation. Neonates rely on non-shivering thermogenesis by the lipolysis of brown adipose tissue and the oxidation of fatty acids in the mitochondria through ATP synthesis [59]. It is reported that neonatal muscles (skeletal muscles) are immature at birth, so shivering thermogenesis is ineffective in producing heat [59].

In altricial species, thermostability is reached until day 18 of life [60], and autonomic thermoregulation is wholly developed at the end of the fourth week [61].

According to the results of the present study, regardless of the mother’s weight, puppies showed a decrease in the temperature from dry to before the intake of colostrum in the anatomical regions of TLBB, TLM, TLE, FPL, and N, and this could be associated with the physiological temperature drop in neonates but also to the limited energy resources that can be compensated through a colostrum intake [62]. Although a control group of non-colostrated puppies was not considered as a comparison for all the experimental groups, the decrease in the temperature observed in the puppies depends on their limited energy reserves immediately after birth [63]. Neonates have minimal glycogen reserves to maintain stable glucose levels, and the immature liver is inefficient at generating energy to thermoregulate when the glycogen stores are emptied, as they are not able to produce heat by shivering, and the brown adipose tissue in newborns is not highly developed [10]. The further increase in the superficial temperature of all the thermal windows after a colostrum intake and at 30 min AB confirm the provided energy source and the impact on the temperature. This effect can be worsened by difficulties in the colostrum intake [63] and may decrease the core temperature and, consequently, the superficial temperature assessed by IRT.

Mila et al. [64] stated that colostrum is an energy source since it can provide 1300–1800 kcal/L and is also high in IgG [65]. However, the variability of the colostrum properties depends on the birth order and suckling time [64], factors not assessed in this study and ones which may be suggested for further research. In general, newborn puppies have a daily energy requirement of approximately 20–26 kcal/100 g of their body weight [61] and need an average colostrum intake of 12 mL per 100 g of their body weight to cover these requirements [65]. In addition, in calves [66], pigs [67], and dogs [68], colostrum contributes to the correct maturation and function of the digestive system and the absorption of nutrients [13]. The amount of glucose that colostrum provides to puppies is crucial because only 1.3% of the body fat content is available in newborns, and hypoglycemia and hypothermia are significant causes of neonatal mortality [64]. Interestingly, the colostrum of small breed bitches (less than 10 kg) provides 10% more energy than the colostrum from large breed females [65], and the ingestion of colostrum could provide a 10% weight recovery during the first 24 h after birth, ensuring the newborn puppy’s survival [13]. This could be why puppy temperatures at 24 h AB reached a certain thermostability between the groups after the colostrum intake, regardless of the dam’s weight, another factor that could intervene is the environmental temperature.

Contrarily to the temperature decrease described in most thermal windows, in T, A, and ULP, there was an increase in the temperature from dry to before the colostrum times. These regions’ anatomical location and vascular irrigation could explain this effect. As reported by various studies, ocular temperature assessed with IRT has shown a positive correlation with the body core temperature compared to other anatomical regions [26,69,70]. The above could be due to the proximity of the ocular orbit to the brain and the ample blood supply provided by the supra and infraorbital arteries [40,71,72,73].

Throughout the seven evaluation times, the significant temperature differences found from wet to 24 h AB in the evaluated regions reflect the disruption of the thermal stability of the newborn puppies and the activation of the thermoregulatory mechanisms. In the intrauterine environment, a heat transfer by maternal circulation maintains the fetal temperature 0.3 to 0.5 °C higher than the mother’s body temperature. However, the maternally dependent puppies experience a rapid temperature loss at birth due to an exposure to the cold extrauterine environment and heat loss by the evaporation from the wet dermal surface by amniotic fluid [59].

Hypothermia is suggested to occur immediately after birth as a protective mechanism to prevent hypoxic damage in the neonate and reduce the metabolic rate to improve the survival of the newborn in the first hours [10,61,74]. This effect could be observed in the newborn puppies of the present study at the first three evaluation times (wet, dry, and colostrum), where the lowest superficial temperatures were recorded. Neonate thermoregulation involves biochemical, anatomical, physiological, and endocrine mechanisms that trigger respiratory and vascular changes and activate the metabolism to produce energy [39,75]. As stated previously, a colostrum intake is essential for newborns to prevent the consequences of hypothermia and reflect the thermal changes in the superficial temperature assessed by IRT.

Another element that influences the temperature differences between the evaluation times is the significant neonatal heat evaporation through the wet dermal surface since animals are born wet [59]. This happens because the insulation and protection function that the coat provides is not efficient since the water (amniotic fluid in this case) has a high thermal conductivity, which generates more significant hypothermia. In addition, other contributing factors include decreased body fat, age, and lack of acclimatization to the environment [76,77,78,79]. In this way, it is possible to explain that body temperatures increased when passing from the wet to dry time, either due to the decrease in the heat loss through evaporation or the decrease in the convection favored by air currents. Another critical factor is the rubbing effect on puppies, which stimulates the dermal vascular microcirculation of the peripheral blood vessels (peripheral vasodilation), increasing microvascular hyperemia at the dermal level.

The thermostability observed at 24 h AB In the puppies from all groups could be attributed to the efficacy of the thermoregulatory response of newborns regardless of the initial weight of the bitch, as shown in the values of IRT in all the thermal windows, where the four groups, when compared to the temperature at wet, had a similar average increase.

Several factors can influence this response, such as the glycogen reserves, colostrum intake, increased digestion, the dam’s presence, and the litter’s members. As observed in the presented results, at 24 h AB, all the puppies reached thermostability after the drying of their coat, colostrum intake, and heat transfer of the mother’s temperature by convection. These thermoregulatory behaviors are important during newborns’ first days since puppies cannot maintain their body temperature when exposed to cold environments up to six days after birth [10]. Therefore, the results suggest that, during the first hours of the life of newborn puppies, their thermoregulation mechanisms are deficient by anatomical and metabolic factors that can be reduced by a colostrum intake, maintaining an adequate body temperature, and its mechanisms to conserve or dissipate heat on the evaluated thermal windows.

One of the main limitations of this study was that since births did not always occur in the same place, the environmental temperatures could not be controlled and standardized for all the cases. It should be clarified that the thermoneutral zone, known as the range of environmental conditions where an animal can regulate heat loss with a minimum of effort [17], should be considered uniformly in puppies to maintain their body temperature within normal limits. Another important point is the great diversity of the breeds and sizes in this species, so the most practical way to group the bitches might be by their weight and not by their breed. Additionally, the pregestational bodyweight of the dams was not recorded in the present study, but this could have given information about the original body condition of the dam before gestation. In some studies, made in humans, positive correlations between maternal obesity and the incidence of high-risk complications at delivery have been described. In this sense, the pre-pregnancy body mass index (BMI) is a major determinant of pregnancy outcome [80]. In addition, it has been seen that a mother’s obesity during pregnancy could predispose the development of obesity in a child between 3 and 5 years old [81]. Therefore, considering the BMI in bitches before and during gestation and its association with the thermal parameters in newborn puppies could be a field for future research.

## 5. Conclusions

In the present study, the IRT reading of the superficial temperature of newborn puppies showed that the weight of the dam seems to influence the neonatal thermostability, where puppies born from bitches of a higher weight presented the highest temperatures from birth to 24 h AB.

Regarding the body regions where temperatures were evaluated, the findings suggest that the thermal windows with the highest temperatures were A, T, N, and ULP, and those with the lowest temperatures were TLBB, TLE, TLM, and FPL. In TLE, there was an interaction between the groups (*p* < 0.0001) and between the groups and times, specifically in G4 where there was an overlap of the temperature values for the time 4 h AB and 24 h AB, as well as for the time 1 h AB and the colostrum time; the other thermal windows had not presented interactions.

One of the most interesting data are that, unlike other studies, this study observed a significant increase in the temperatures of the puppies in the thermal windows A, T, and ULP immediately after ingesting colostrum, which could be an area for future research. An important aspect to note is that the use of the thermal windows used in this work in newborn puppies had not been reported previously, so further studies using these thermal windows in this species could be considered. Similarly, the concern arises to know how much the weight of newborn puppies can influence their ability to achieve thermostability.

## Figures and Tables

**Table 1:** Mean temperature (°C) of thoracic limb brachial biceps (TLBB) window at different times in newborn puppies from bitches classified into 4 groups according to its weight.

Time	G_1_ *n* = 47	G_2_ *n* = 68	G_3_ *n* = 79	G_4_ *n* = 96
Wet	27.43 ± 0.065 ^c,5^	27.76 ± 0.051 ^b,6^	27.80 ± 0.046 ^b,6^	28.29 ± 0.042 ^a,6^
Dry	28.27 ± 0.120 ^b,3,4^	28.61 ± 0.114 ^b,3,4^	28.65 ± 0.114 ^b,3,4^	29.19 ± 0.096 ^a,3,4^
Colostrum	27.75 ± 0.057 ^c,4,5^	28.11 ± 0.047 ^b,5^	28.20 ± 0.049 ^b,5^	28.72 ± 0.045 ^a,5^
30 min AB	28.03 ± 0.104 ^c,4^	28.33 ± 0.093 ^b,c,4,5^	28.40 ± 0.075 ^b,4,5^	28.91 ± 0.073 ^a,4,5^
1 h AB	28.53 ± 0.094 ^c,3^	28.86 ± 0.078 ^b,3^	28.95 ± 0.075 ^b,3^	29.47 ± 0.063 ^a,3^
4 h AB	29.43 ± 0.110 ^c,2^	29.85 ± 0.096 ^b,2^	29.91 ± 0.082 ^b,2^	30.38 ± 0.076 ^a,2^
24 h AB	30.59 ± 0.103 ^c,1^	30.95 ± 0.083 ^b,1^	30.95 ± 0.070 ^b,1^	31.45 ± 0.068 ^a,1^

(ANOVA, StatSoft Inc. 8.0, Tulsa, Oakland, CA, USA). *n*, number of puppies; weight of dams according to category: G_1_, 4–8 kg; G_2_, 8.1–16 kg; G_3_, 16.1–32 kg; G_4_, 32.1–39.6 kg; AB, after birth; least-squares mean ± standard error. *p* values for the different temperatures of the pups between groups and times in the regions evaluated were <0.0001 in all cases. ^a,b,c^ Different superscripts among columns indicate significant temperature differences between dam’s weight groups at the same time. ^1,2,3,4,5,6^ Different numbers among rows indicate significant differences between times in the same dam’s weight group.

**Table 2:** Mean temperature (°C) of thoracic limb elbow (TLE) window at different times in newborn puppies from bitches classified into 4 groups according to its weight.

Time	G_1_ *n* = 47	G_2_ *n* = 68	G_3_ *n* = 79	G_4_ *n* = 96
Wet	27.11 ± 0.057 ^c,5^	27.30 ± 0.051 ^b,c,7^	27.42 ± 0.047 ^b,7^	28.01 ± 0.039 ^a,4^
Dry	30.78 ± 0.074 ^c,4^	30.96 ± 0.058 ^b,c,4^	31.11 ± 0.058 ^b,4^	31.40 ± 0.060 ^a,3^
Colostrum	30.29 ± 0.067 ^c,3^	30.52 ± 0.048 ^b,c,5^	30.64 ± 0.049 ^b,5^	30.87 ± 0.078 ^a,2^
30 min AB	31.04 ± 0.059 ^c,4^	31.23 ± 0.046 ^b,c,3^	31.34 ± 0.044 ^a,b,3^	31.57 ± 0.086 ^a,3^
1 h AB	30.04 ± 0.071 ^c,3^	30.22 ± 0.061 ^b,c,6^	30.36 ± 0.060 ^b,6^	30.84 ± 0.055 ^a,2^
4 h AB	32.13 ± 0.068 ^b,2^	32.33 ± 0.057 ^a,b,2^	32.47 ± 0.059 ^a,2^	32.56 ± 0.078 ^a,1^
24 h AB	32.81 ± 0.073 ^a,b,1^	33.05 ± 0.066 ^b,1^	33.12 ± 0.059 ^b,1^	32.62 ± 0.094 ^a,1^

(ANOVA, StatSoft Inc. 8.0, Tulsa, Oakland, CA, USA). *n*, number of puppies; weight of dams according to category: G_1_, 4–8 kg; G_2_, 8.1–16 kg; G_3_, 16.1–32 kg; G_4_, 32.1–39.6 kg; AB, after birth; least-squares mean ± standard error. *p* values for the different temperatures of the pups between groups and times in the regions evaluated were <0.0001 in all cases. ^a,b,c^ Different superscripts among columns indicate significant temperature differences between the dam’s weight groups at the same time. ^1,2,3,4,5,6,7^ Different numbers among rows indicate significant differences between times in the same dam’s weight group.

**Table 3:** Mean temperature (°C) of thoracic limb metacarpals (TLM) window at different times in newborn puppies from bitches classified into 4 groups according to its weight.

Time	G_1_ *n* = 47	G_2_ *n* = 68	G_3_ *n* = 79	G_4_ *n* = 96
Wet	26.98 ± 0.073 ^c,5^	27.14 ± 0.061 ^b,c,5^	27.30 ± 0.054 ^b,5^	27.75 ± 0.056 ^a,6^
Dry	29.84 ± 0.073 ^c,2,3^	30.02 ± 0.062 ^b,c,2,3^	30.19 ± 0.062 ^b,2^	30.46 ± 0.063 ^a,4^
Colostrum	28.96 ± 0.043 ^c,4^	29.16 ± 0.033 ^b,4^	29.25 ± 0.036 ^b,4^	29.71 ± 0.031 ^a,7^
30 min AB	29.43 ± 0.10 ^b,3,4^	29.63 ± 0.078 ^b,3^	29.73 ± 0.081 ^b,3^	30.14 ± 0.072 ^a,5^
1 h AB	30.24 ± 0.063 ^c,1,2^	30.38 ± 0.058 ^b,c,2^	30.49 ± 0.053 ^b,2^	30.95 ± 0.047 ^a,3^
4 h AB	30.08 ± 0.33 ^b,2^	30.37 ± 0.24 ^b,2^	30.46 ± 0.20 ^b,2^	31.19 ± 0.052 ^a,2^
24 h AB	30.83 ± 0.10 ^b,1^	30.89 ± 0.091 ^b,1^	31.04 ± 0.083 ^b,1^	31.54 ± 0.073 ^a,1^

(ANOVA, StatSoft Inc. 8.0, Tulsa, Oakland, CA, USA). *n*, number of puppies; weight of dams according to category: G_1_, 4–8 kg; G_2_, 8.1–16 kg; G_3_, 16.1–32 kg; G_4_, 32.1–39.6 kg; AB, after birth; least-squares mean ± standard error. *p* values for the different temperatures of the pups between groups and times in the regions evaluated were <0.0001 in all cases. ^a,b,c^ Different superscripts among columns indicate significant temperature differences between the dam’s weight groups at the same time. ^1,2,3,4,5,6,7^ Different numbers among rows indicate significant differences between times in the same dam’s weight group.

**Table 4:** Mean temperature (°C) of thoracic (T) window at different times in newborn puppies from bitches classified into 4 groups according to its weight.

Time	G_1_ *n* = 47	G_2_ *n* = 68	G_3_ *n* = 79	G_4_ *n* = 96
Wet	28.79 ± 0.18 ^a,6^	28.80 ± 0.19 ^a,6^	29.06 ± 0.13 ^a,5^	29.20 ± 0.13 ^a,5^
Dry	31.44 ± 0.049 ^c,5^	31.64 ± 0.042 ^b,5^	31.69 ± 0.040 ^b,4^	31.93 ± 0.035 ^a,4^
Colostrum	31.66 ± 0.074 ^b,4,5^	31.87 ± 0.056 ^b,4,5^	31.86 ± 0.052 ^b,4^	32.16 ± 0.047 ^a,4^
30 min AB	31.99 ± 0.052 ^c,3,4^	32.17 ± 0.039 ^b,3,4^	32.26 ± 0.037 ^b,3^	32.51 ± 0.033 ^a,2^
1 h AB	32.12 ± 0.063 ^c,2,3^	32.35 ± 0.052 ^b,2,3^	32.38 ± 0.045 ^b,2,3^	32.62 ± 0.041 ^a,2^
4 h AB	32.40 ± 0.066 ^c,2^	32.68 ± 0.054 ^b,2^	32.63 ± 0.059 ^b,c,2^	32.92 ± 0.050 ^a,2^
24 h AB	33.22 ± 0.054 ^c,1^	33.38 ± 0.052 ^b,c,1^	33.49 ± 0.045 ^b,1^	33.72 ± 0.041 ^a,1^

(ANOVA, StatSoft Inc. 8.0, Tulsa, Oakland, CA, USA). *n*, number of puppies; weight of dams according to category: G_1_, 4–8 kg; G_2_, 8.1–16 kg; G_3_, 16.1–32 kg; G_4_, 32.1–39.6 kg; AB, after birth; least-squares mean ± standard error. *p* values for the different temperatures of the pups between groups and times in the regions evaluated were <0.0001 in all cases. ^a,b,c^ Different superscripts among columns indicate significant temperature differences between the dam’s weight groups at the same time. ^1,2,3,4,5,6^ Different numbers among rows indicate significant differences between times in the same dam’s weight group.

**Table 5:** Mean temperature (°C) of abdominal (A) window at different times in newborn puppies from bitches classified into 4 groups according to its weight.

Time	G_1_ *n* = 47	G_2_ *n* = 68	G_3_ *n* = 79	G_4_ *n* = 96
Wet	29.47 ± 0.12 ^b,6^	29.77 ± 0.10 ^a,b,5^	29.76 ± 0.098 ^a,b,5^	29.95 ± 0.085 ^a,5^
Dry	32.23 ± 0.046 ^c,5^	32.49 ± 0.037 ^b,4^	32.55 ± 0.037 ^b,4^	32.75 ± 0.032 ^a,4^
Colostrum	32.46 ± 0.075 ^b,4,5^	32.66 ± 0.062 ^b,4^	32.59 ± 0.060 ^b,4^	32.90 ± 0.054 ^a,4^
30 min AB	32.75 ± 0.073 ^b,3,4^	32.99 ± 0.061 ^b,3^	32.96 ± 0.060 ^b,3^	33.22 ± 0.055 ^a,3^
1 h AB	32.84 ± 0.086 ^b,3^	33.09 ± 0.072 ^a,b,3^	33.08 ± 0.063 ^a,b,3^	33.30 ± 0.061 ^a,3^
4 h AB	33.20 ± 0.083 ^b,2^	33.46 ± 0.068 ^a,b,2^	33.38 ± 0.070 ^b,2^	33.63 ± 0.063 ^a,2^
24 h AB	33.74 ± 0.061 ^c,1^	33.92 ± 0.060 ^b,c,1^	34.02 ± 0.053 ^b,1^	34.23 ± 0.047 ^a,1^

(ANOVA, StatSoft Inc. 8.0, Tulsa, Oakland, CA, USA). *n*, number of puppies; weight of dams according to category: G_1_, 4–8 kg; G_2_, 8.1–16 kg; G_3_, 16.1–32 kg; G_4_, 32.1–39.6 kg; AB, after birth; least-squares mean ± standard error. *p* values for the different temperatures of the pups between groups and times in the regions evaluated were <0.0001 in all cases. ^a,b,c^ Different superscripts among columns indicate significant temperature differences between the dam’s weight groups at the same time. ^1,2,3,4,5,6^ Different numbers among rows indicate significant differences between times in the same dam’s weight group.

**Table 6:** Mean temperature (°C) of femoral pelvic limb (FPL) window at different times in newborn puppies from bitches classified into 4 groups according to its weight.

Time	G_1_ *n* = 47	G_2_ *n* = 68	G_3_ *n* = 79	G_4_ *n* = 96
Wet	27.54 ± 0.071 ^c,6^	27.90 ± 0.057 ^b,6^	27.97 ± 0.051 ^b,5^	28.29 ± 0.046 ^a,5^
Dry	28.49 ± 0.12 ^b,3,4^	28.83 ± 0.11 ^b,3,4^	28.84 ± 0.11 ^b,3^	29.24 ± 0.095 ^a,3^
Colostrum	27.86 ± 0.065 ^c,5,6^	28.25 ± 0.057 ^b,5,6^	28.39 ± 0.062 ^b,4^	28.72 ± 0.055 ^a,4^
30 min AB	28.15 ± 0.12 ^b,4,5^	28.54 ± 0.10 ^b,4,5^	28.51 ± 0.087 ^b,4^	28.89 ± 0.085 ^a,4^
1 h AB	28.61 ± 0.097 ^c,3^	28.97 ± 0.084 ^b,3^	29.03 ± 0.077 ^b,3^	29.40 ± 0.066 ^a,3^
4 h AB	29.62 ± 0.082 ^c,2^	30.12 ± 0.071 ^b,2^	30.08 ± 0.064 ^b,2^	30.43 ± 0.061 ^a,2^
24 h AB	30.70 ± 0.11 ^c,1^	31.06 ± 0.088 ^b,c,1^	31.15 ± 0.087 ^b,1^	31.46 ± 0.075 ^a,1^

(ANOVA, StatSoft Ic. 8.0, Tulsa, Oakland, CA, USA). *n*, number of puppies; weight of dams according to category: G_1_, 4–8 kg; G_2_, 8.1–16 kg; G_3_, 16.1–32 kg; G_4_, 32.1–39.6 kg; AB, after birth; least-squares mean ± standard error. *p* values for the different temperatures of the pups between groups and times in the regions evaluated were <0.0001 in all cases. ^a,b,c^ Different superscripts among columns indicate significant temperature differences between the dam’s weight groups at the same time. ^1,2,3,4,5,6^ Different numbers among rows indicate significant differences between times in the same dam’s weight group.

**Table 7:** Mean temperature (°C) of nasal (N) window at different times in newborn puppies from bitches classified into 4 groups according to its weight.

Time	G_1_ *n* = 47	G_2_ *n* = 68	G_3_ *n* = 79	G_4_ *n* = 96
Wet	28.09 ± 0.079 ^c,3^	28.53 ± 0.069 ^b,3^	28.64 ± 0.061 ^b,3^	29.05 ± 0.055 ^a,3^
Dry	29.56 ± 0.067 ^c,1,2^	30.02 ± 0.059 ^b,1,2^	30.07 ± 0.053 ^b,1,2^	30.52 ± 0.046 ^a,1,2^
Colostrum	29.32 ± 0.090 ^c,1,2^	29.79 ± 0.081 ^b,2^	29.89 ± 0.072 ^b,2^	30.28 ± 0.065 ^a,2^
30 min AB	29.20 ± 0.098 ^c,2^	29.65 ± 0.088 ^b,2^	29.75 ± 0.080 ^b,2^	30.17 ± 0.070 ^a,2^
1 h AB	29.31 ± 0.18 ^b,1,2^	29.66 ± 0.15 ^b,2^	29.81 ± 0.14 ^a,b,2^	30.21 ± 0.12 ^a,2^
4 h AB	29.20 ± 0.19 ^c,2^	29.64 ± 0.15 ^b,c,2^	29.89 ± 0.14 ^a,b,2^	30.35 ± 0.12 ^a,2^
24 h AB	29.75 ± 0.14 ^c,1^	30.26 ± 0.11 ^b,1^	30.35 ± 0.097 ^a,b,1^	30.68 ± 0.093 ^a,1^

(ANOVA, StatSoft Inc. 8.0, Tulsa, Oakland, CA, USA). *n*, number of puppies; weight of dams according to category: G_1_, 4–8 kg; G_2_, 8.1–16 kg; G_3_, 16.1–32 kg; G_4_, 32.1–39.6 kg; AB, after birth; least-squares mean ± standard error. *p* values for the different temperatures of the pups between groups and times in the regions evaluated were <0.0001 in all cases. ^a,b,c^ Different superscripts among columns indicate significant temperature differences between the dam’s weight groups at the same time. ^1,2,3^ Different numbers among rows indicate significant differences between times in the same dam’s weight group.

**Table 8:** Mean temperature (°C) of upper left palpebral (ULP) window at different times in newborn puppies from bitches classified into 4 groups according to its weight.

Time	G_1_ *n* = 47	G_2_ *n* = 68	G_3_ *n* = 79	G_4_ *n* = 96
Wet	30.69 ± 0.079 ^b,5^	30.78 ± 0.066 ^b,5^	30.83 ± 0.060 ^b,5^	31.09 ± 0.058 ^a,5^
Dry	31.56 ± 0.097 ^b,4^	31.67 ± 0.084 ^a,b,4^	31.68 ± 0.081 ^a,b,4^	31.95 ± 0.075 ^a,4^
Colostrum	32.00 ± 0.089 ^b,3^	32.01 ± 0.077 ^b,3^	32.07 ± 0.068 ^b,3^	32.39 ± 0.062 ^a,3^
30 min AB	32.88 ± 0.063 ^b,2^	32.92 ± 0.054 ^b,2^	32.99 ± 0.049 ^b,2^	33.30 ± 0.045 ^a,2^
1 h AB	32.85 ± 0.048 ^c,2^	32.93 ± 0.040 ^b,c,2^	33.03 ± 0.036 ^b,2^	33.29 ± 0.032 ^a,2^
4 h AB	33.02 ± 0.095 ^b,2^	33.01 ± 0.079 ^b,2^	33.16 ± 0.073 ^b,2^	33.48 ± 0.069 ^a,2^
24 h AB	33.62 ± 0.066 ^b,1^	33.65 ± 0.050 ^b,1^	33.79 ± 0.048 ^b,1^	34.07 ± 0.044 ^a,1^

(ANOVA, StatSoft Inc. 8.0, Tulsa, Oakland, CA, USA). *n*, number of puppies; weight of dams according to category: G_1_, 4–8 kg; G_2_, 8.1–16 kg; G_3_, 16.1–32 kg; G_4_, 32.1–39.6 kg; AB, after birth; least-squares mean ± standard error. *p* values for the different temperatures of the pups between groups and times in the regions evaluated were <0.0001 in all cases. ^a,b,c^ Different superscripts among columns indicate significant temperature differences between the dam’s weight groups at the same time. ^1,2,3,4,5^ Different numbers among rows indicate significant differences between times in the same dam’s weight group.

**Table 9:** Significant correlations between the thoracic limb brachial biceps (TLBB) temperatures of newborn puppies and the weight of the dams at different times: wet (with amniotic fluid), dry (once the puppy was rubbered), colostrum (after colostrum intake and separated from mother’s teat), 30 min AB, 1 h AB, 4 h AB, and 24 h AB.

Variables	Correlation Coefficient (*r*)	*p*-Value
Wet	0.540	<0.0001
Dry	0.301	<0.0001
Colostrum	0.601	<0.0001
30 min AB	0.399	<0.0001
1 h AB	0.435	<0.0001
4 h AB	0.369	<0.0001
24 h AB	0.365	<0.0001

Spearman’s rank correlation coefficients and their statistical significance between the dam’s weight and temperature of puppies at different times. AB: after birth.

**Table 10:** Significant correlations between the weight of the dam and the superficial temperature at thoracic limb elbow (TLE) thermal window in newborn puppies at different times: wet (with amniotic fluid), dry (once the puppy was rubbered), colostrum (after colostrum intake and separated from mother’s teat), 30 min AB, 1 h AB, 4 h AB, and 24 h AB.

Variables	Correlation Coefficient (*r*)	*p*-Value
Wet	0.610	<0.0001
Dry	0.380	<0.0001
Colostrum	0.326	<0.0001
30 min AB	0.286	<0.0001
1 h AB	0.484	<0.0001
4 h AB	0.222	<0.001
24 h AB	−0.177	0.0026

Spearman’s rank correlation coefficients and their statistical significance between the dam’s weight and temperature of puppies at different times. AB: after birth.

**Table 11:** Significant correlations between the weight of the dam and the superficial temperature at thoracic limb metacarpals (TLM) window in newborn puppies at different times: wet (with amniotic fluid), dry (once the puppy was rubbered), colostrum (after colostrum intake and separated from mother’s teat), 30 min AB, 1 h AB, 4 h AB, and 24 h AB.

Variables	Correlation Coefficient (*r*)	*p*-Value
Wet	0.475	<0.0001
Dry	0.358	<0.0001
Colostrum	0.634	<0.0001
30 min AB	0.334	<0.0001
1 h AB	0.485	<0.0001
4 h AB	0.241	<0.0001
24 h AB	0.342	<0.0001

Spearman’s rank correlation coefficients and their statistical significance between the dam’s weight and temperature of puppies at different times. AB: after birth.

**Table 12:** Significant correlations in the T thermal window temperatures of newborn puppies and the weight of the dams at different times: wet (with amniotic fluid), dry (once the puppy was rubbered), colostrum (after colostrum intake and separated from mother’s teat), 30 min AB, 1 h AB, 4 h AB, and 24 h AB.

Variables	Correlation Coefficient (*r*)	*p*-Value
Wet	0.127	0.031
Dry	0.429	<0.0001
Colostrum	0.316	<0.0001
30 min AB	0.467	<0.0001
1 h AB	0.342	<0.0001
4 h AB	0.286	<0.0001
24 h AB	0.391	<0.0001

Spearman’s rank correlation coefficients and their statistical significance between the dam’s weight and temperature of puppies at different times. AB: after birth.

## Data Availability

Data are contained within the article.

## References

[1] Mota-Rojas D., López A., Martínez-Burnes J., Muns R., Villanueva-García D., Mora-Medina P., González-Lozano M., Olmos-Hernández A., Ramírez-Necoechea R. (2018). Is vitality assessment important in neonatal animals?. CAB Rev. Perspect. Agric. Vet. Sci. Nutr. Nat. Resour..

[2] Dwyer C.M. (2014). Maternal behaviour and lamb survival: From neuroendocrinology to practical application. Animal.

[3] Ocepek M., Andersen I.L. (2017). What makes a good mother? Maternal behavioural traits important for piglet survival. Appl. Anim. Behav. Sci..

[4] Lezama-García K., Mariti C., Mota-Rojas D., Martínez-Burnes J., Barrios-García H., Gazzano A. (2019). Maternal behaviour in domestic dogs. Int. J. Vet. Sci. Med..

[5] Madani T., Allouche L., Saffidine N., Kaouane N., Belkasmi F., Semara L. (2013). Maternal and neonatal behaviors of Ouled Djellal sheep breed and their effects on production parameters. Small Rumin. Res..

[6] Mellor D. (2019). Preparing for Life after Birth: Introducing the Concepts of Intrauterine and Extrauterine Sensory Entrainment in Mammalian Young. Animals.

[7] Lévy F., Keller M., Poindron P. (2004). Olfactory regulation of maternal behavior in mammals. Horm. Behav..

[8] Mota-Rojas D., Orihuela A., Strappini-Asteggiano A., Nelly Cajiao-Pachón M., Agüera-Buendía E., Mora-Medina P., Ghezzi M., Alonso-Spilsbury M. (2018). Teaching animal welfare in veterinary schools in Latin America. Int. J. Vet. Sci. Med..

[9] Veronesi M. (2016). Assessment of canine neonatal viability-the Apgar score. Reprod. Domest. Anim..

[10] Münnich A., Küchenmeister U. (2014). Causes, diagnosis and therapy of common diseases in neonatal puppies in the first days of life: Cornerstones of practical approach. Reprod. Domest. Anim..

[11] Uchańska O., Ochota M., Eberhardt M., Niżański W. (2022). Dead or alive? A review of perinatal factors that determine canine neonatal viability. Animals.

[12] Villanueva-García D., Mota-Rojas D., Martínez-Burnes J., Olmos-Hernández A., Mora-Medina P., Salmerón C., Gómez J., Boscato L., Gutiérrez-Pérez O., Cruz V. (2021). Hypothermia in newly born piglets: Mechanisms of thermoregulation and pathophysiology of death. J. Anim. Behav. Biometeorol..

[13] Reyes-Sotelo B., Ogi A., Mora-Medina P., Mariti C., Olmos-Hernández A., Hernández-Ávalos I., Domínguez-Oliva A., Rosas M.E., Verduzco-Mendoza A., Gazzano A. (2022). Early Blood Analysis and Gas Exchange Monitoring in the Canine Neonate: Effect of Dam’s Size and Birth Order. Animals.

[14] Nord A., Nilsson J.F., Sandell M.I., Nilsson J.-Å. (2009). Patterns and dynamics of rest-phase hypothermia in wild and captive blue tits during winter. J. Comp. Physiol. B.

[15] Terrien J., Perret M., Aujard F. (2011). Behavioral thermoregulation in mammals: A review. Front. Biosci..

[16] Mota-Rojas D., Titto C.G., Orihuela A., Martínez-Burnes J., Gómez-Prado J., Torres-Bernal F., Flores-Padilla K., Carvajal-de la Fuente V., Wang D., la Fuente V.C. (2021). Physiological and behavioral mechanisms of thermoregulation in mammals. Animals.

[17] Lezama-García K., Mota-Rojas D., Pereira A.M.F., Martínez-Burnes J., Ghezzi M., Domínguez A., Gómez J., de Mira Geraldo A., Lendez P., Hernández-Ávalos I. (2022). Transient Receptor Potential (TRP) and thermoregulation in animals: Structural biology and neurophysiological aspects. Animals.

[18] Nowak R., Poindron P. (2006). From birth to colostrum: Early steps leading to lamb survival. Reprod. Nutr. Dev..

[19] Vannucchi C.I.C., Rodrigues J.J.A., Silva L.L.C.G., Lúcio C.C.F., Veiga G.A.L.G. (2012). A clinical and hemogasometric survey of neonatal lambs. Small Rumin. Res..

[20] Traas A.M. (2008). Resuscitation of canine and feline neonates. Theriogenology.

[21] Wilborn R.R. (2018). Small animal neonatal health. Vet. Clin. N. Am. Small Anim. Pract..

[22] Indrebø A., Trangerud C., Moe L. (2007). Canine neonatal mortality in four large breeds. Acta Vet. Scand..

[23] Nakamura K., Morrison S.F. (2011). Central efferent pathways for cold-defensive and febrile shivering. J. Physiol..

[24] Fisher A.D., Morton R., Dempsey J.M.A., Henshall J.M., Hill J.R. (2008). Evaluation of a new approach for the estimation of the time of the LH surge in dairy cows using vaginal temperature and electrodeless conductivity measurements. Theriogenology.

[25] Sevegnani K.B., Fernandes D.P.B., da Silva S.H.M.-G. (2016). Evaluation of thermorregulatory capacity of dairy buffaloes using infrared thermography. Eng. Agrícola.

[26] Travain T., Colombo E.S., Heinzl E., Bellucci D., Prato Previde E., Valsecchi P. (2015). Hot dogs: Thermography in the assessment of stress in dogs (Canis familiaris)—A pilot study. J. Vet. Behav..

[27] McMillan T., Spaulding K., Digangi B.A., Cussen V.A., Reid P.J., Collinds K.A. (2022). Handling Shelter Dogs. Animal Behavior for Shelter Veterinarians and Staff.

[28] Casas-Alvarado A., Mota-Rojas D., Hernández-Ávalos I., Mora-Medina P., Olmos-Hernández A., Verduzco-Mendoza A., Reyes-Sotelo B., Martínez-Burnes J. (2020). Advances in infrared thermography: Surgical aspects, vascular changes, and pain monitoring in veterinary medicine. J. Therm. Biol..

[29] Bertoni A., Mota-Rojas D., Álvarez-Macias A., Mora-Medina P., Guerrero-Legarreta I., Morales-Canela A., Gómez-Prado J., José-Pérez N., Martínez-Burnes J. (2020). Scientific findings related to changes in vascular microcirculation using infrared thermography in the river buffalo. J. Anim. Behav. Biometeorol..

[30] Mota-Rojas D., Titto C.G., de Mira Geraldo A., Martínez-Burnes J., Gómez J., Hernández-Ávalos I., Casas A., Domínguez A., José N., Bertoni A. (2021). Efficacy and function of feathers, hair, and glabrous skin in the thermoregulation strategies of domestic animals. Animals.

[31] Gama L.T., Dickerson G.E., Young L.D., Leymaster K.A. (1991). Effects of breed, heterosis, age of dam, litter size, and birth weight on lamb mortality1. J. Anim. Sci..

[32] Dwyer C.M., Morgan C.A. (2006). Maintenance of body temperature in the neonatal lamb: Effects of breed, birth weight, and litter size1. J. Anim. Sci..

[33] Tuchscherer M., Puppe B., Tuchscherer A., Tiemann U. (2000). Early identification of neonates at risk: Traits of newborn piglets with respect to survival. Theriogenology.

[34] Kirkden R.D., Broom D.M., Andersen I.L. (2013). Invited review: Piglet mortality: Management solutions. J. Anim. Sci..

[35] Darwish R.A., Abou-Ismail U.A., El-Kholya S.Z. (2010). Differences in post-parturient behaviour, lamb performance and survival rate between purebred Egyptian Rahmani and its crossbred Finnish ewes. Small Rumin. Res..

[36] Groppetti D., Ravasio G., Bronzo V., Pecile A. (2015). The role of birth weight on litter size and mortality within 24 h of life in purebred dogs: What aspects are involved?. Anim. Reprod. Sci..

[37] Mila H., Grellet A., Feugier A., Chastant-Maillard S. (2015). Differential impact of birth weight and early growth on neonatal mortality in puppies1,2. J. Anim. Sci..

[38] Veronesi M.C., Panzani S., Faustini M., Rota A. (2009). An Apgar scoring system for routine assessment of newborn puppy viability and short-term survival prognosis. Theriogenology.

[39] Reyes-Sotelo B., Mota-Rojas D., Mora-Medina P., Ogi A., Mariti C., Olmos-Hernández A., Martínez-Burnes J., Hernández-Ávalos I., Sánchez-Millán J., Gazzano A. (2021). Blood biomarker profile alterations in newborn canines: Effect of the mother’s weight. Animals.

[40] Casas-Alvarado A., Martínez-Burnes J., Mora-Medina P., Hernández-Avalos I., Domínguez-Oliva A., Lezama-García K., Gómez-Prado J., Mota-Rojas D. (2022). Thermal and circulatory changes in diverse body regions in dogs and cats evaluated by infrared thermography. Animals.

[41] World Small Animal Veterinary Association (WSAVA) Global Nutritional Assessment Guidelines. http://wsava.org/wp-content/uploads/2020/01/Global-Nutritional-Assesment-Guidelines-Spanish.pdf.

[42] Tønnessen R., Borge K.S., Nødtvedt A., Indrebø A. (2012). Canine perinatal mortality: A cohort study of 224 breeds. Theriogenology.

[43] Federation Cynologigue Internationale (FCI). http://www.fci.be.

[44] Eneroth A., Linde-Forsberg C., Uhlhorn M., Hall M. (1999). Radiographic pelvimetry for assessment of dystocia in bitches: A clinical study in two terrier breeds. J. Small Anim. Pract..

[45] Kammersgaard T.S., Malmkvist J., Pedersen L.J. (2013). Infrared thermography—A noninvasive tool to evaluate thermal status of neonatal pigs based on surface temperature. Animal.

[46] Sherwin C.M., Christiansen S.B., Duncan I.J., Erhard H.W., Lay D.C., Mench J.A., O’Connor C.E., Petherick J.C. (2003). Guidelines for the ethical use of animals in applied ethology studies. Appl. Anim. Behav. Sci..

[47] Piccione G., Fazio F., Giudice E., Refinetti R. (2009). Body size and the daily rhythm of body temperature in dogs. J. Therm. Biol..

[48] Rigotti C.F., Jolliffe C.T., Leece E.A. (2015). Effect of prewarming on the body temperature of small dogs undergoing inhalation anesthesia. J. Am. Vet. Med. Assoc..

[49] Kwon C.J., Brundage C.M. (2019). Quantifying body surface temperature differences in canine coat types using infrared thermography. J. Therm. Biol..

[50] Jordan M., Bauer A.E., Stella J.L., Croney C. Temperature Requirements for Dogs. https://www.extension.purdue.edu/extmedia/va/va-16-w.pdf.

[51] Schrank M., Mollo A., Contiero B., Romagnoli S. (2019). Bodyweight at Birth and growth rate during the neonatal period in three canine breeds. Animals.

[52] Veronesi M.C., Faustini M., Probo M., Rota A., Fusi J. (2022). Refining the APGAR score cutoff values and viability classes according to breed body size in newborn dogs. Animals.

[53] Harri M., Mononen J., Haapanen K., Korhonen H. (1991). Postnatal changes in hypothermic response in farmborn blue foxes and raccoon dogs. J. Therm. Biol..

[54] Trangerud C., Grøndalen J., Indrebø A., Tverdal A., Ropstad E., Moe L. (2007). A longitudinal study on growth and growth variables in dogs of four large breeds raised in domestic environments1. J. Anim. Sci..

[55] Tattersall G.J. (2016). Infrared thermography: A non-invasive window into termal physiology. Comp. Biol. Physiol..

[56] Travain T., Colombo E.S., Grandi L.C., Heinzl E., Pelosi A., Prato Previde E., Valsecchi P. (2016). How good is this food? A study on dogs’ emotional responses to a potentially pleasant event using infrared thermography. Physiol. Behav..

[57] Nitrini A.G.C., Cogliati B., Matera J.M. (2021). Thermographic assessment of skin and soft tissue tumors in cats. J. Feline Med. Surg..

[58] Waddell R.E., Marino D.J., Loughin C.A., Tumulty J.W., Dewey C.W., Sackman J. (2015). Medical infrared thermal imaging of cats with hyperthyroidism. Am. J. Vet. Res..

[59] Asakura H. (2004). Fetal and neonatal thermoregulation. J. Nippon Med. Sch..

[60] Pineda M., Dooley M. (2008). McDonald’s Veterinary Endocrinology and Reproduction.

[61] Lawler D. (2008). Neonatal and pediatric care of the puppy and kitten. Theriogenology.

[62] Le Dividich J., Noblet J. (1981). Colostrum intake and thermoregulation in the neonatal pig in relation to environmental temperature. Neonatology.

[63] Mugnier A., Chastant S., Saegerman C., Gaillard V., Grellet A., Mila H. (2021). Management of low birth weight in canine and feline species: Breeder profiling. Animals.

[64] Mila H., Feugier A., Grellet A., Anne J., Gonnier M., Martin M., Rossig L., Chastant-Maillard S. (2015). Immunoglobulin G concentration in canine colostrum: Evaluation and variability. J. Reprod. Immunol..

[65] Chastant-Maillard S., Aggouni C., Albaret A., Fournier A., Mila H. (2017). Canine and feline colostrum. Reprod. Domest. Anim..

[66] Bühler C., Hammon H., Rossi G.L., Blum J.W. (1998). Small intestinal morphology in eight-day-old calves fed colostrum for different durations or only milk replacer and treated with long-R3-insulin-like growth factor I and growth hormone. J. Anim. Sci..

[67] Burrin D.G., Shulman R.J., Reeds P.J., Davis T.A., Gravitt K.R. (1992). Porcine Colostrum and milk stimulate visceral organ and skeletal muscle protein synthesis in neonatal piglets. J. Nutr..

[68] Schwarz S.M., Heird W.C. (1994). Effects of feeding on the small intestinal mucosa of beagle pups during the first 5 d of life. Am. J. Clin. Nutr..

[69] Bartolomé E., Sánchez M.J., Molina A., Schaefer A.L., Cervantes I., Valera M. (2013). Using eye temperature and heart rate for stress assessment in young horses competing in jumping competitions and its possible influence on sport performance. Animal.

[70] Church J.S., Hegadoren P.R., Paetkau M.J., Miller C.C., Regev-Shoshani G., Schaefer A.L., Schwartzkopf-Genswein K.S. (2014). Influence of environmental factors on infrared eye temperature measurements in cattle. Res. Vet. Sci..

[71] Stewart M., Webster J.R., Verkerk G.A., Schaefer A.L., Colyn J.J., Stafford K.J. (2007). Non-invasive measurement of stress in dairy cows using infrared thermography. Physiol. Behav..

[72] Sutherland M.A., Worth G.M., Dowling S.K., Lowe G.L., Cave V.M., Stewart M. (2020). Evaluation of infrared thermography as a non-invasive method of measuring the autonomic nervous response in sheep. PLoS ONE.

[73] Kim S.-M., Cho G.-J. (2021). Validation of eye temperature assessed using infrared thermography as an indicator of welfare in horses. Appl. Sci..

[74] Lezama-García K., Mota-Rojas D., Martínez-Burnes J., Villanueva-García D., Domínguez-Oliva A., Gómez-Prado J., Mora-Medina P., Casas-Alvarado A., Olmos-Hernández A., Soto P. (2022). Strategies for hypothermia compensation in altricial and precocial newborn mammals and their monitoring by infrared thermography. Vet. Sci..

[75] Hillman N.H., Kallapur S.G., Jobe A.H. (2012). Physiology of transition from intrauterine to extrauterine life. Clin. Perinatol..

[76] Mallet M.L. (2002). Pathophysiology of accidental hypothermia. QJM.

[77] Oncken A., Kirby R., Rudloff E. (2001). Hypotherma in critically ill dogs and cats. Compend. Contin. Educ. Pract. Vet..

[78] Dwyer C.M., Conington J., Corbiere F., Holmøy I.H., Muri K., Nowak R., Rooke J., Vipond J., Gautier J.M. (2016). Invited review: Improving neonatal survival in small ruminants: Science into practice. Animal.

[79] Kozat S. (2018). Hypothermia in newborn calves. J. Istanb. Vet. Sci..

[80] Nelson S.M., Matthews P., Poston L. (2010). Maternal metabolism and obesity: Modifiable determinants of pregnancy outcome. Hum. Reprod. Update.

[81] Griffiths L.J., Hawkins S.S., Cole T.J., Dezateux C., Millennium Cohort Study Child Health Group (2010). Risk factors for rapid weight gain in preschool children: Findings from UK-wide prospective study. Int. J. Obes..

